# Uncovering the Structural Diversity of Y(III) Naphthalene-2,6-Dicarboxylate MOFs Through Coordination Modulation

**DOI:** 10.3389/fchem.2019.00036

**Published:** 2019-01-31

**Authors:** Sarah L. Griffin, Claire Wilson, Ross S. Forgan

**Affiliations:** ^1^WestCHEM School of Chemistry, University of Glasgow, Glasgow, United Kingdom; ^2^EPSRC Centre for Innovative Manufacturing in Continuous Manufacturing and Crystallisation, University of Strathclyde Technology and Innovation Centre, Glasgow, United Kingdom

**Keywords:** metal-organic frameworks, yttrium, coordination modulation, microwave synthesis, fluorescent sensing

## Abstract

Metal-organic frameworks (MOFs)—network structures built from metal ions or clusters and connecting organic ligands—are typically synthesized by solvothermal self-assembly. For transition metal based MOFs, structural predictability is facilitated by control over coordination geometries and linker connectivity under the principles of isoreticular synthesis. For rare earth (RE) MOFs, coordination behavior is dominated by steric and electronic factors, leading to unpredictable structures, and poor control over self-assembly. Herein we show that coordination modulation—the addition of competing ligands into MOF syntheses—offers programmable access to six different Y(III) MOFs all connected by the same naphthalene-2,6-dicarboxylate ligand, despite controlled synthesis of multiple phases from the same metal-ligand combination often being challenging for rare earth MOFs. Four of the materials are isolable in bulk phase purity, three are amenable to rapid microwave synthesis, and the fluorescence sensing ability of one example toward metal cations is reported. The results show that a huge variety of structurally versatile MOFs can potentially be prepared from simple systems, and that coordination modulation is a powerful tool for systematic control of phase behavior in rare earth MOFs.

## Introduction

Research revolving around metal-organic frameworks (MOFs)—network structures wherein metal ions or clusters are connected by organic linkers into diverse topologies (Furukawa et al., 2013)—has rapidly increased in recent years following publication of landmark materials in the late 1990s (Chui et al., 1999; Li et al., 1999), with there being ~70,000 MOF structures reported in the Cambridge Structural database as of 2016 (Moghadam et al., 2017). The reason for this interest can be attributed to the permanent porosity and vast range of structural and chemical properties that can be imparted on MOFs, leading to an array of applications from gas storage and separation (Yang and Xu, 2017) to drug delivery and biosensing (Abánades Lázaro and Forgan, 2019). As more MOFs are discovered it is becoming apparent that there is a large range of fascinating topologies accessible, showing diversities in their coordination, solvation, and porosity.

A considerable amount of research focuses on the discovery and synthesis of new frameworks, utilizing a wide range of metals—most commonly transition metals—and organic linkers. We have studied the modulated self-assembly of Zr(IV) (Marshall et al., 2016) and Sc(III) (Marshall et al., 2018) MOFs in detail, yet studies into the synthesis of new yttrium materials appear to be somewhat limited. Whilst yttrium is a *d* block metal, it is considered as a rare earth (RE) metal and displays coordination chemistry similar to the lanthanides when in the Y(III) oxidation state. As such, there are several reports of lanthanide doped Y-MOFs, for example MIL-78 (Serre et al., 2004) and MIL-92 (Surblé et al., 2005), with the possibility of utilizing the inherent luminescence of europium and terbium to impart nitroaromatic detection and tunable photoluminescence properties on the otherwise non-luminescent materials (Singha et al., 2015; Zheng et al., 2015). Of the single metal Y-MOFs reported, several exhibit permanent porosity and high thermal and water stability, leading to a range of potential applications (Weng et al., 2006; Luo et al., 2008; Jiang et al., 2010; Gong et al., 2012; Duan et al., 2013; Kim et al., 2015; Bezrukov and Dietzel, 2017; Mohideen et al., 2018). For example, a 1,2,4,5-tetrakis(4-carboxyphenyl)benzene based Y-MOF has been utilized for indoor moisture control (Abdulhalim et al., 2017), whilst a framework based on a hexa-carboxylate linker showed selective adsorption of C_2_H_2_ and CO_2_ over CH_4_ (Liu et al., 2017).

**Figure 1:**
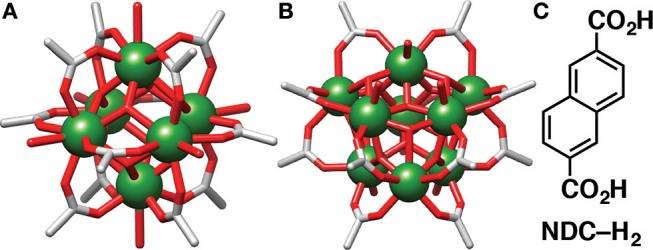
Examples of 12-connected **(A)** [Y_6_(OH)_8_(RCO_2_)_12_(H_2_O)_6_]^2−^ and **(B)** [Y_9_(O)_2_(OH)_14_ (RCO_2_)_12_(H_2_O)_7_]^3−^ SBUs accessible through coordination modulation. **(C)** Chemical structure of the naphthalene-2,6-dicarboxylic acid (NDC-H_2_) linker.

Much like in the synthesis of many other MOFs, coordination modulation—the addition of monotopic linkers as capping agents or crystallization promotors (McGuire and Forgan, 2015)—has been used in the synthesis of Y-MOFs in order to achieve highly crystalline material. Coordination modulation is routinely implemented in the synthesis of MOFs, most commonly enhancing crystallinity and allowing the isolation of single crystals, although under certain conditions, coordination modulation can also control particle size, allowing for the synthesis of nanomaterials (Guo et al., 2012; Chen et al., 2018), or introduce defects throughout a structure whilst maintaining the overall topology (Wu et al., 2013). It should be noted that the use of different modulators in syntheses does not tend to have an effect on the topology of the resultant material. For example, in the case of Zr-MOFs of the UiO topology, the use of different modulators, such as L-proline, benzoic and acetic acid, still results in the formation of the UiO structure, merely with different physical or chemical properties (Schaate et al., 2011; Marshall et al., 2016). Unlike their transition element counter parts however, RE elements have less predictable coordination geometries, resulting in a greater variety of coordination geometries. As a result there is much more difficulty in the prediction of rare earth MOF structures compared to transition metal-based MOFs (Pagis et al., 2016), with coordination modulation potentially perturbing self-assembly to allow isolation of new structures (Decadt et al., 2012). For example, Eddaoudi et al. have introduced 2-fluorobenzoic acid as an efficient modulator of RE MOFs, isolating for example Y-MOFs containing hexanuclear (Figure 1A) (Xue et al., 2013, 2015; Luebke et al., 2015) and nonanuclear (Figure 1B) (Guillerm et al., 2014; Abdulhalim et al., 2017; Chen et al., 2017) secondary building units (SBUs).

Herein, we report an extensive study into the effects of coordination modulation on the crystallization of naphthalene-2,6-dicarboxylic acid (NDC-H_2_, Figure 1C) Y-MOFs. Contrary to many modulated syntheses in which modulation does not affect overall topology, in this case the use of different modulators led to the crystallization of a range of phases containing Y(III) and 2,6-NDC^2−^, each exhibiting different coordination, solvation, and void space. The selected modulators ranged in pH, size and functionality. Several of the Y-MOFs were synthesized in bulk with phase purity via solvothermal synthesis, with multiple syntheses also successfully being carried out via microwave assisted heating, considerably reducing synthesis time. The luminescent properties of one of the MOFs were also examined, studying the sensing abilities of the framework in the presence of a variety of metals ions.

## Results and Discussion

Use of different modulators allowed isolation of six different framework structures wherein Y^3+^ cations are linked by 2,6-NDC^2−^ units (see SI, section Synthesis). A summary of synthetic conditions is given in Table 1.

**Table 1:** Summary of synthetic conditions used to prepare single crystals of Y-NDC MOFs **1**–**6**.

**MOF**	**Y source**	**Modulator**	**Solvent**	**T**	**Time**
**1**	YCl_3_·6H_2_O*^*a*^*	None	DMF	120°C	24 h*^*b*^*
**2**	Y(NO_3_)_3_·6H_2_O	H_2_O (2 equiv)	DMF	120°C	24 h
**3**	Y(NO_3_)_3_·6H_2_O	HNO_3_ (16 equiv)	DMF	120°C/16°C	24 h/72 h
**4**	Y(NO_3_)_3_·6H_2_O	Acetic Acid (40 equiv)	DMF	120°C	24 h*^*c*^*
**5**	Y(NO_3_)_3_·6H_2_O	2-Fluorobenzoic acid (8 equiv)/HNO_3_ (40 equiv)	DMF/H_2_O (5:1)	120°C	2 h*^*c*^*
**6**	Y(NO_3_)_3_·6H_2_O	Tartaric acid (0.5 equiv)	DMF/H_2_O/EtOH (2:1:1)	80°C	24 h

a *Can also be synthesized using Y(NO_3_)_3_·6H_2_O*.

b *Alternatively 1 h in a microwave reactor*.

c *Alternatively 2 h in a microwave reactor*.

**Figure 2:**
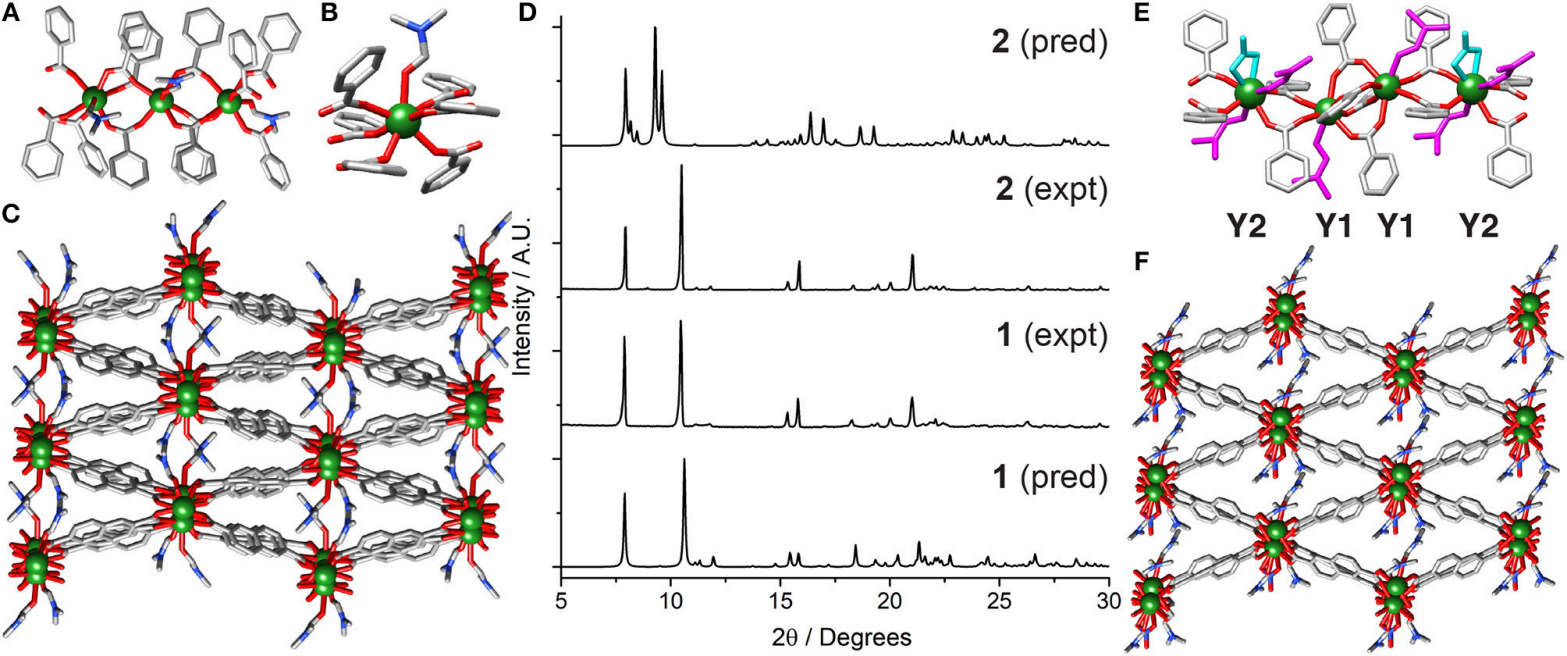
Crystal structure of **1**. **(A)** The one-dimensional chain SBU. **(B)** The coordination sphere of Y1. **(C)** The overall packing structure showing potential porosity. **(D)** Stacked PXRD patterns of bulk samples of **1** and **2** compared to their predicted patterns, confirming crystals of **2** were isolated from material with bulk composition of **1**. Crystal structure of **2**. **(E)** One-dimensional chain SBU with coordinating nitrate (cyan) and DMF (magenta). **(F)** Packing structure showing structural relationship with **1**. H atoms and disorder removed for clarity in all crystal structure images.

Subjecting YCl_3_ and 2,6-NDC-H_2_ to solvothermal synthesis in DMF at 120°C resulted in the isolation of **[Y**_**2**_**(NDC)**_**3**_**(C**_**3**_**H**_**7**_**NO)**_**2**_**]**_***n***_ **(1)**_._ **1** is a 3D coordination polymer, which crystallizes in the monoclinic space group P2_1_/c. The asymmetric unit contains two crystallographically independent Y^3+^ ions having the same connectivity, three 2,6-NDC^2−^ linkers and two DMF molecules. One dimensional chains of Y^3+^ cations running down the crystallographic *c* axis (Figure 2A) are connected by carboxylate units of the linkers, with three NDC^2−^ units bridging adjacent metal ions in the (η^1^:η^1^:μ_2_) motif seen in the related Sc_2_(BDC)_3_ MOF (Miller et al., 2005; Perles et al., 2005). The larger size of Y^3+^ compared to Sc^3+^ leads to coordination of one DMF molecule, making the Y^3+^ molecules seven-coordinate (Figure 2B), and distancing them further from one another along the chain (Y···Y ~4.8 Å) than in Sc_2_(BDC)_3_ (Sc···Sc ~4.4 Å). The chains connect into a net with diamond-shaped pores into which the DMF molecules protrude (Figure 2C).

The synthesis of **1** can be successfully scaled up to produce the material in bulk with high crystallinity and phase purity, as shown by powder X-ray diffraction (PXRD), which was carried out on a washed and dried sample, showing a close match to the pattern predicted from the crystal structure (Figure 2D). Rapid microwave synthesis (Lin et al., 2012) of **1** is also possible. Yttrium chloride was used rather than yttrium nitrate in order to produce single crystals suitable for structure determination X-ray diffraction, however the same structure is produced when using yttrium nitrate (Figure S1).

In a synthesis using Y(NO_3_)·6H_2_O and NDC-H_2_ doped with two additional equivalents of water, it was possible to isolate crystals of **[Y**_**3**_**(NDC)**_**4**_**(C**_**3**_**H**_**7**_**NO)**_**4**_**(NO**_**3**_**)]**_***n***_ **(2)**, from a bulk solid that was shown by PXRD to comprise almost entirely **1** (Figure 2D and Figure S2). Structure **2** crystallizes in the triclinic space group $P\bar{1}$, with the asymmetric unit containing one and a half Y^3+^ ions, two 2,6-NDC^2−^ linkers, one and a half coordinated nitrate molecules and two coordinated DMF molecules. The unit cell contains two crystallographically independent Y^3+^ centers, one of which is disordered across two crystallographically equivalent positions through an inversion center. The structure is related to **1**; it is comprised of 1D chains of single metal ions, repeating in a [Y1···Y1···Y2]_*n*_ fashion over the independent Y^3+^ ions (Figure 2E). The Y1 centers are linked by four bridging NDC^2−^ units with carboxylate groups in the (η^1^:η^1^:μ_2_) motif, with each Y1 linked to an adjacent Y2 by two carboxylates in the same (η^1^:η^1^:μ_2_) motif and the seven-coordinate geometry completed by a DMF solvent molecule. As such, the Y2 centers coordinate to four carboxylate oxygen atoms (two bridging from each Y1 center) with the coordination sphere filled by two DMF molecules and one bidentate nitrate anion, making it eight-coordinate. The Y2 ions are disordered across an inversion center, resulting in two distinct positions 1.3213(3) Å apart with associated disorder of the coordinated ligands. Along the chain, which has the sequence Y1Y1Y2Y1Y1Y2, the Y1···Y1 distance is 4.1635(1) Å, while the Y1···Y2 distances are longer and asymmetric, with one Y1···Y2 distance of 5.1434(2) Å and the other of 5.4725(2) Å, likely because Y1···Y1 are connected via four carboxylate groups from four linkers whereas Y1···Y2 are connected by only two carboxylate groups from two linkers. The overall structure looks similar to **1**, with diamond-like pores containing DMF and nitrate ligands pointing into potential pore space (Figure 2F).

**Figure 3:**
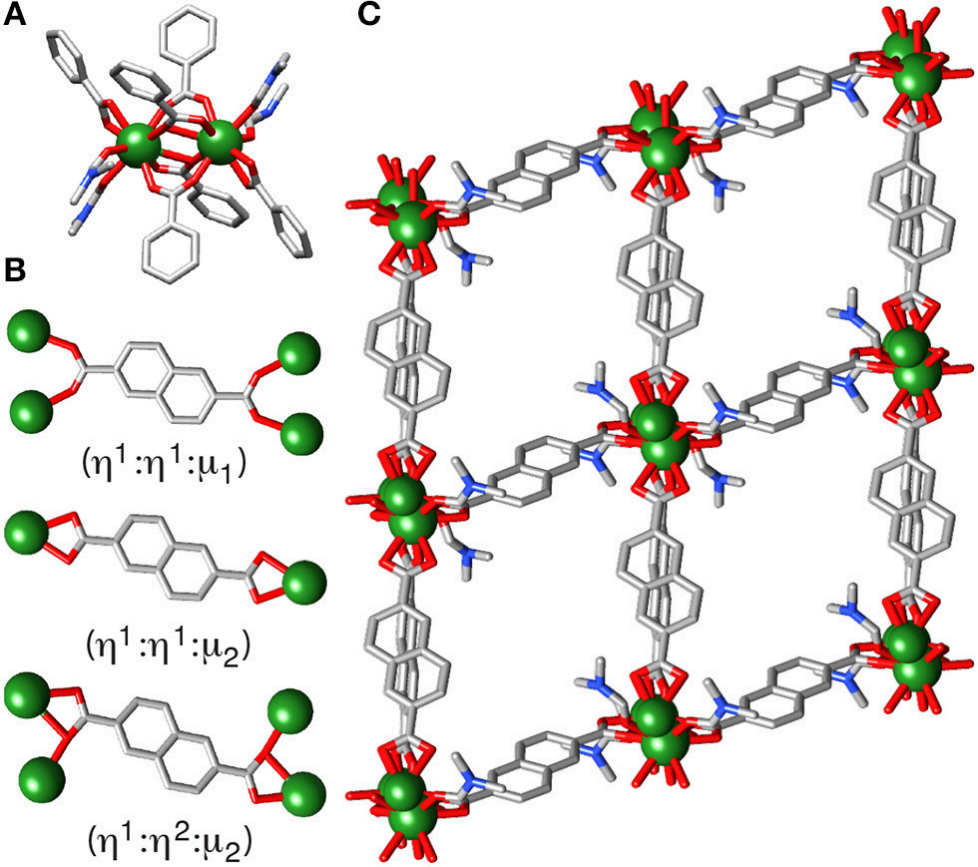
The crystal structure of **3**. **(A)** The dimeric SBU. **(B)** The differing coordination modes of the crystallographically independent NDC^2−^ ligands. **(C)** The packing structure viewed down the *a* axis. H atoms removed for clarity.

Attempts to produce phase pure samples of **2**, by deliberate addition of 16 equivalents of nitric acid as a source of nitrate into solvothermal syntheses, resulted in the isolation of **[Y**_**2**_**(NDC)**_**3**_**(C**_**3**_**H**_**7**_**NO)**_**4**_**]**_***n***_ **(3)**, which has a significantly different structure but contains no nitrate. The structure, which crystallizes in the triclinic $P\bar{1}$ space group, has discrete dimeric SBUs (Figure 3A) rather than 1D chains of Y^3+^ ions. The asymmetric unit consists of a nine-coordinate Y^3+^ ion, one and a half 2,6-NDC^2−^ linkers and two coordinated DMF molecules. Four NDC^2−^ linkers bridge the binuclear SBU, two with the (η^1^:η^1^:μ_2_) motif and two with the (η^1^:η^2^:μ_2_) motif, with adjacent pairs of Y^3+^ ions in the SBU separated by 3.9933(2) Å and related by an inversion center. The Y^3+^ ions also coordinate to a linker in a terminal bidentate (η^1^:η^2^:μ_1_) motif (Figure 3B) and two DMF molecules, making them nine-coordinate. The bridging linkers form square grid-like sheets along the *bc* plane, with the terminal linkers connecting the sheets in an offset manner to form a 3D net with a calculated void space [N_2_ probe, Mercury 3.10.3 (Macrae et al., 2008)] of 31.9%, with continuous channels running through the framework along the *a* axis (Figure 3C). A discrete complex, [Y_2_(3,5-DHB)_2_(CH_3_CO_2_)_4_(H_2_O)_4_] (DHB = dihydroxybenzoate), with a closely related coordination arrangement to the dimeric SBU in **3**, has been observed previously (Dan et al., 2006).

The synthesis of **3** differs from the others in that crystallization occurs at room temperature after the reaction solution has been heated to 120°C for 24 h. Upon removing the reaction jar from the oven, the clear yellow solution (previously colorless) was left undisturbed in a chilled room for a further 3 days, wherein crystallization of block crystals occurred. The reaction was successfully scaled up, proven both visually and by SCXRD, however the framework is not stable to solvent loss as demonstrated by a change in PXRD pattern, highlighting the decomposition (Figure S3). Structurally analogous MOFs have been prepared from Ce, Eu, and Tb, which also decompose in air (Wang et al., 2002), although a Nd derivative has shown sufficient stability to allow gas uptake, with S_BET_ = 150 m^2^g^−1^ (Wang et al., 2015).

**Figure 4:**
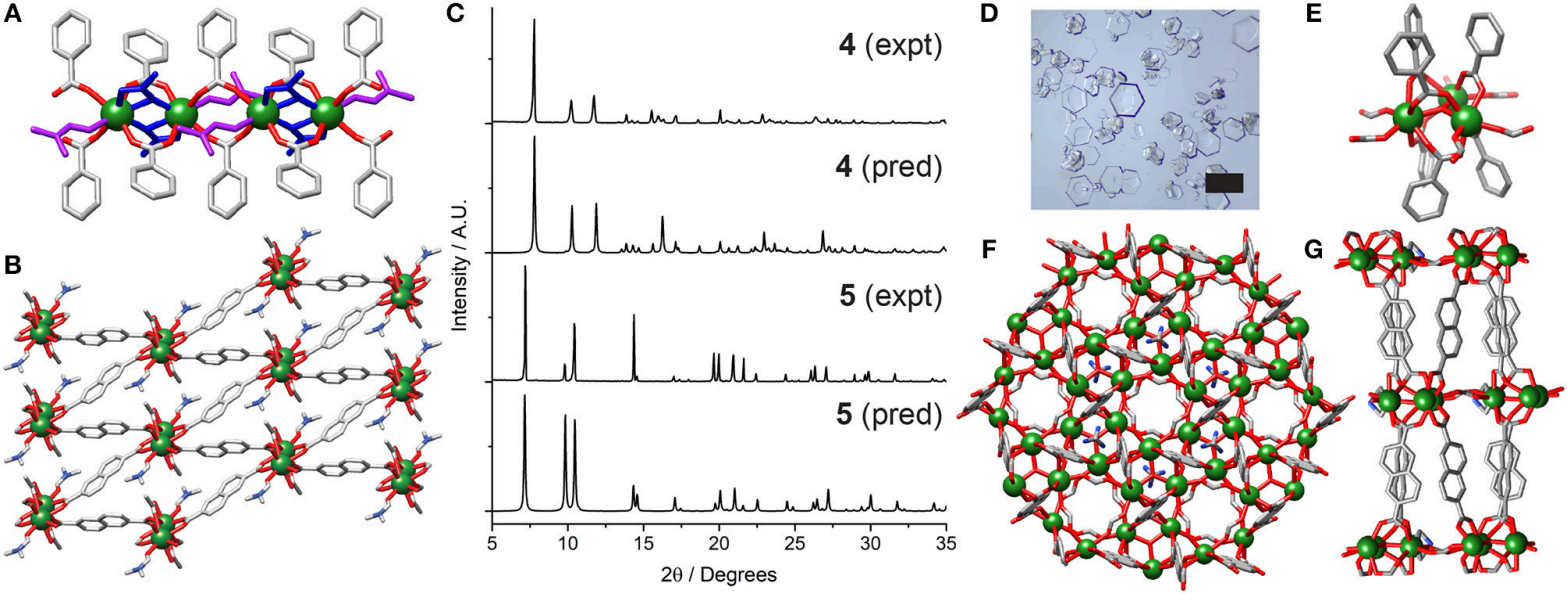
Crystal structure of **4**. **(A)** One dimensional chain SBU, with coordinated acetate (blue) and DMF (magenta). **(B)** Packing structure viewed down the *b* axis. **(C)** Stacked PXRD patterns of **4** and **5** compared to their predicted patterns. **(D)** Optical image of the hexagonal plates of **5**, which give rise to preferred orientation related peak height discrepancies in the PXRD pattern of bulk samples. Scale bar 500 μm. Crystal structure of **5**. **(E)** Trigonal SBU showing bridging formates. **(F)** Packing structure showing solvent inaccessible voids between SBUs. **(G)** Hexagonal packing arrangement viewed down the *c* axis.

Subsequently, carboxylate-based modulators were examined. Incorporation of 40 equivalents of acetic acid into the synthesis resulted in the isolation of **[Y(NDC)(CH**_**3**_**CO**_**2**_**)(C**_**3**_**H**_**7**_**NO)]**_***n***_ **(4)** as a phase pure material. **4** crystallizes in the triclinic space group $P\bar{1}$, with the asymmetric unit containing an eight-coordinate Y^3+^ ion, one 2,6-NDC^2−^ linker, a coordinated acetate and a DMF molecule. The structure is related to **1**, with one-dimensional chains of Y^3+^ ions bridged by NDC^2−^ linkers, but one NDC^2−^ ligand is replaced by two acetate ligands (Figure 4A). The structure can be thought of as containing chains of dimers; two equivalent Y^3+^ ions, related by an inversion center, are bridged by carboxylate units from two NDC^2−^ ligands in the (η^1^:η^1^:μ_2_) motif and two acetates in the (η^1^:η^2^:μ_2_) motif. These dimers are linked to one another by two further NDC^2−^ carboxylates, also in the (η^1^:η^1^:μ_2_) motif, with the Y^3+^ coordination sphere being completed by a DMF molecule. The Y···Y distance within the “dimer” is 3.8689(9) Å, while the Y···Y distance to the adjacent “dimer” in the chain is 5.570(1) Å. **4** has a similar packing arrangement to **1**, with diamond-shaped pores down the *b* axis filled with DMF molecules (Figure 4B). Unlike **2**, the sample can be prepared in bulk, with phase purity confirmed by PXRD (Figure 4C).

The combination of 2-fluorobenzoic acid, nitric acid, and water as co-modulators for RE MOFs has previously resulted in isolation of MOFs with UiO-66 (Xue et al., 2013) and MIL-88 (Wei et al., 2017) nets, as well as novel topologies (Guillerm et al., 2014). With Y^3+^ and NDC^2−^, a structure related to MIL-88C is formed, **(CH**_**3**_**)**_**2**_**NH**_**2**_**[Y**_**3**_**(NDC)**_**3**_**(HCOO)**_**3**_**(OH)]**_***n***_ **(5)**. Structure **5** crystallizes as hexagonal plates (Figure 4D) in the $P\bar{3}$ space group, with the asymmetric unit containing one seven-coordinate Y^3+^ ion, one 2,6-NDC^2−^ linker, one formate and one third of a dimethylammonium ion (both generated by decomposition of DMF), and one third of a hydroxyl ion. The framework is comprised of [Y_3_(μ_3_-OH)(RCO_2_)_6_] trimeric SBUs [Figure 4E, Y1···O1C = 2.2986(3) Å] linked by NDC^2−^ linkers into the MIL-88C topology (Figure 4F). The SBUs, which lie in the *ab* plane, are further connected via two bridging formates per Y^3+^ ion to form a 2D sheet; the distance between neighboring clusters, measured between the oxygen atoms of adjacent bridging μ_3_-OH ligands (O1C), is 10.376(2) Å. Disordered dimethylammonium ions sit in the plane of the clusters, forming NH···O hydrogen bonds to oxygen atoms of the linking formates [N1S···O1F = 3.0180(5) Å] and NDC^2−^ linkers [N1S···O1 = 3.0100(3) Å]. The overall structural formula can be confirmed by ^1^H NMR spectroscopic analysis of digested samples, clearly showing the presence of dimethylammonium cations and formate anions with integral ratios consistent with the overall formula (Figure S6). The structure has pockets of void space totaling 20.3% of the framework, with the bridging formates preventing a continuous solvent accessible pore (Figure 4G). Whilst similar to MIL-88C type structures, the higher coordination number of Y compared to transition metals results in the additional coordination of the formate groups to link the SBUs, expected to limit the breathing nature of **5**, a property associated with transition metal linked MIL-88C materials (Horcajada et al., 2011). The crystal structure of the analogous Er-MOF has recently been reported, with the Y-MOF identified by PXRD (Wei et al., 2017).

**Figure 5:**
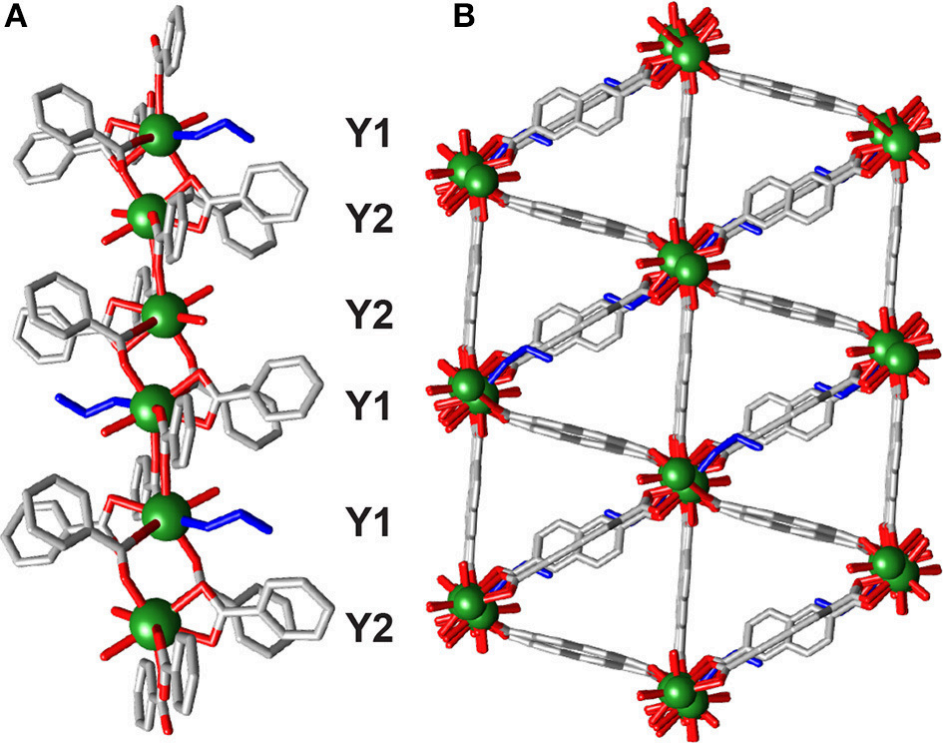
The crystal structure of **6**. **(A)** One dimensional chain SBU, with coordinating ethanol colored blue. **(B)** Packing structure showing significant void space, with ordered DMF guests removed for clarity.

Tartaric acid has previously been shown to be an efficient modulator in the synthesis of a mixed metal Y-BDC MOF in a mixed EtOH/water solvent system (Abdelbaky et al., 2014), and so conditions (heating to 80°C for 24 h) inspired by this were implemented with Y^3+^ and NDC^2−^. Single crystals of **[Y**_**2**_**(NDC)**_**3**_**(C**_**2**_**H**_**5**_**OH)(H**_**2**_**O)**_**3**_**]**_***n***_**·3(C**_**3**_**H**_**7**_**NO) (6)** could be isolated from the resulting gel. **6** crystallizes in the triclinic space group $P\bar{1}$; the crystal structure was determined from a selected crystal and does not represent the bulk material, which, after washing and drying, PXRD analysis confirms to be predominantly **1** (Figure S7). The asymmetric unit of **6** contains two Y^3+^ ions, three 2,6-NDC^2−^ linkers, a coordinated ethanol molecule and three coordinated water molecules. Chains of single Y^3+^ ions run along the *bc* plane throughout the framework (Figure 5A), alternating in a -Y1-Y1-Y2-Y2- fashion. Each Y^3+^ is bridged by two NDC^2−^ carboxylates in the (η^1^:η^1^:μ_2_) motif and is also bridged by a bidentate NDC^2−^ carboxylate in a (η^1^:η^1^:μ_1_) arrangement. The difference between Y1 and Y2 lies in coordinated solvents; Y1 is coordinated to one water molecule and one ethanol molecule, whilst Y2 is coordinated to two water molecules. Within the linear metal chain there are three different but similar Y-Y distances—Y1···Y1 = 4.742(2) Å, Y2···Y2 = 4.731(2) Å, and Y1···Y2 = 4.8846(2) Å,—highlighting the similarity of the eight-coordinate Y^3+^ ions.

The chains are connected in an approximately hexagonal manner by the NDC^2−^ ligands to form a framework with a 29% void space [N_2_ probe, Mercury 3.10.3 (Macrae et al., 2008)], running in disconnected sheets along the *bc* plane (Figure 5B) and containing ordered DMF solvent molecules. It was unfortunately not possible to prepare a phase-pure sample of **6**.

By simple modification of synthetic conditions, six different MOFs have been characterized from a single metal-ligand system, with counterions, solvents, and most importantly modulators playing key roles in directing structure formation. Of the six, the syntheses of frameworks **1**, **4**, and **5** were successfully scaled up to produce the materials in bulk with high crystallinity and phase purity, as shown by PXRD which was carried out on washed and dried samples. The predicted and experimental powder patterns show good overlap, while minor discrepancies in peak intensities can be attributed to preferential orientation of the packed powder samples. These three frameworks were also successfully produced through microwave-assisted synthesis. Each was carried out on the same scale as that of the bulk solvothermal synthesis and produced crystalline, phase pure material in 2 h and under compared to 24 h solvothermally (Figures S4 and S5). Compounds **2** and **6** crystallize alongside **1** under specific conditions, while **3** can also be scaled up, but appears to be unstable to drying.

**Figure 6:**
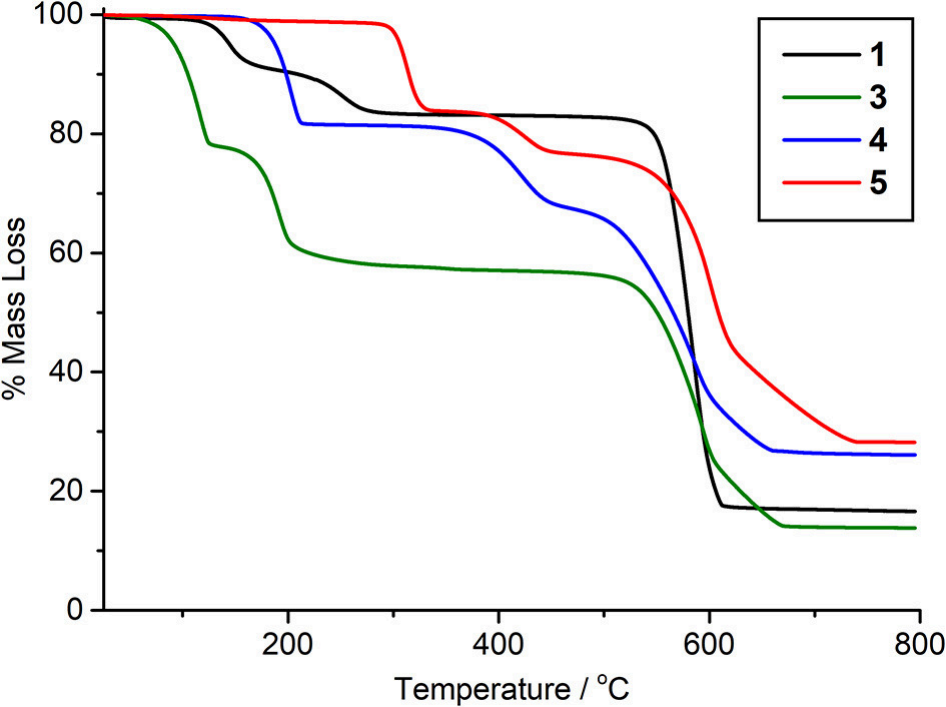
Thermogravimetric analysis in air of **1**, **3**, **4**, and **5**.

**1**, **4**, and **5** can be produced in bulk and have potential voids if solvents can be successfully removed. As such, thermogravimetric analysis was carried out on bulk samples (Figures S8 and S9), along with an undried sample of **3**, which is unstable to solvent removal, to assess its bulk composition (Figure 6). The TGA profile of **1** shows the stepwise loss of its two DMF molecules, with a mass loss of 8.6% followed by another of 7.5% between 115°C and 290°C. The final weight loss of 66.2% between 525°C and 618°C can be attributed to the organic linkers. Analysis of **4** shows a similar initial loss of coordinated DMF between 154°C and 214°C, followed by a stepped 55.3% mass loss between 340°C and 670°C arising from the loss of acetate and linkers. **5** shows a first weight loss of 16.2% between 287°C and 336°C which closely correlates with the loss of dimethylammonium cations and formate anions. The following two-step loss of 55.5% can be attributed to the loss of NDC^2−^ linkers and hydroxide. Due to decomposition of **3** on solvent removal, the material was not fully dried before analysis. As such, the initial 22.1% weight loss between 55°C and 126°C arises from encapsulated DMF. The subsequent mass loss of 20.2% correlates with the loss of coordinated DMF, and is followed by a 43.7% mass loss due to organic linkers, confirming the bulk composition suggested by the single crystal structure.

Despite the ability to remove solvent molecules from the materials, none were found to be porous to N_2_ at 77 K. Removal of coordinated DMF from **1** and **4** presumably results in framework collapse, although it is noted that during the peer review period, a Ce(III) analog of **1** was reported which could be desolvated at 300°C to a phase that was porous to CO_2_ (Atzori et al., in press). For **5**, whilst the framework does show void space, it is possible that access to the void channels is blocked due to charge-balancing dimethylammonium ions lying in the cluster layer of the framework.

**Figure 7:**
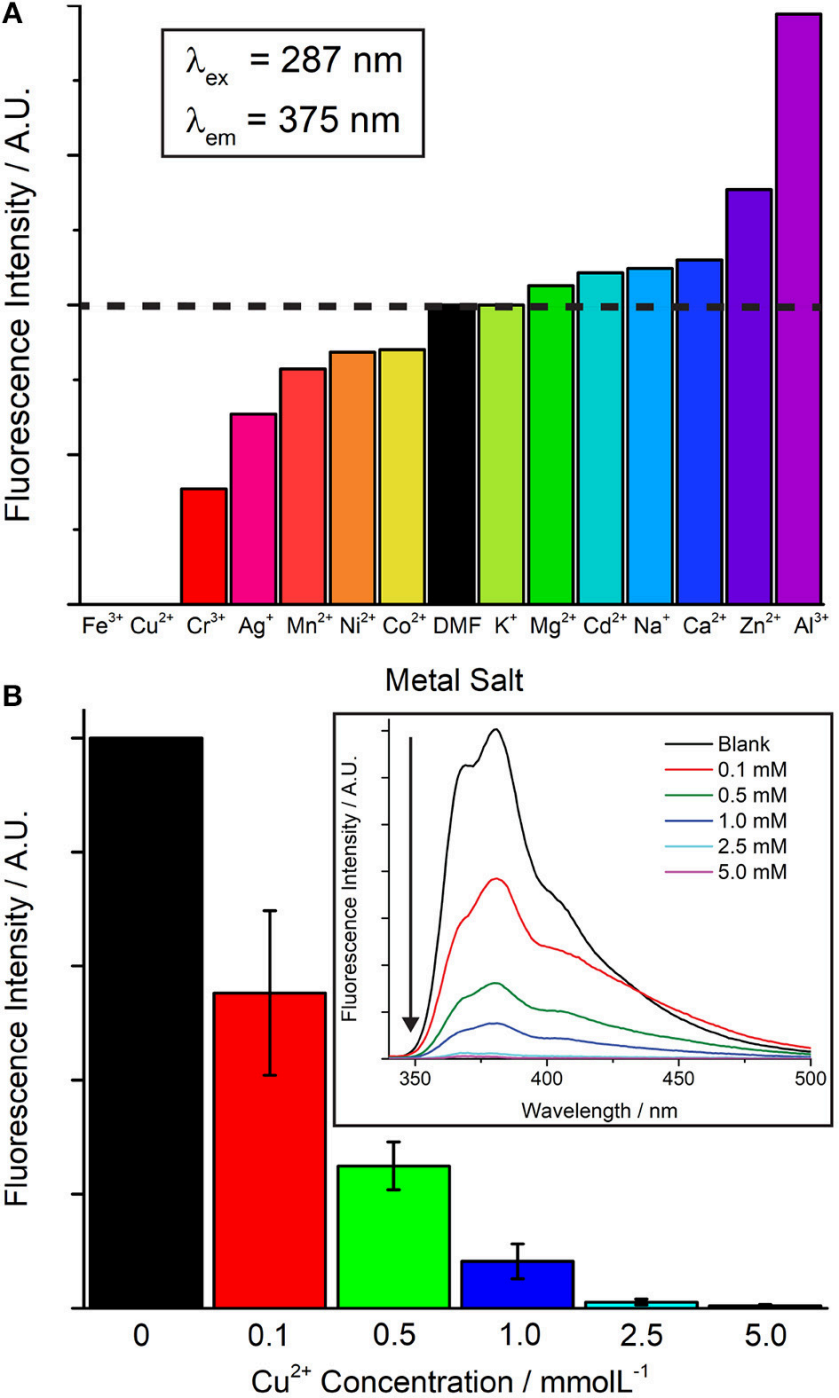
**(A)** Changes in luminescence of samples of **5** when contacted with 5 mM DMF solutions of metal nitrates. **(B)** Concentration dependence of turn-off sensing of Cu^2+^. Experiments carried out in triplicate, typical spectra for Cu^2+^ sensing shown as an inset.

The luminescence properties of **5** were investigated in DMF solution in the presence of a variety of metal nitrates, with differing extents of cation exchange with the dimethylammonium ions a possible mechanism for fluorescent sensing. The excitation of the free NDC-H_2_ linker at λ = 287 nm leads to an emission maximum at λ = 375 nm, with a shoulder at λ = 364 nm, dictating the excitation wavelength for **5**. Finely ground framework **5** (2 mg) was immersed in 5 ml of 5 mM DMF solutions of M^x+^(NO_3_)_x_ (M = Ag^+^, Al^3+^, Cd^2+^, Ca^2+^, Cr^3+^, Co^2+^, Cu^2+^, Fe^3+^, Mn^2+^, Mg^2+^, Ni^2+^, K^+^, Na^+^, Zn^2+^) and sonicated for 30 min. The luminescence of **5** can be seen to vary to some degree in the presence of all the metal nitrates (Figure 7A). Soaking in Fe^3+^ and Cu^2+^ solutions led to complete quenching of luminescence, whereas Al^3+^ and Zn^2+^ solutions gave rise to a considerable increase in luminescence. The presence of Cd^2+^ and Zn^2+^ notably leads to a shift in the intensity ratio between the 364 and 375 nm peaks, along with the appearance of a new shoulder peak at 350 nm (Figure S10).

To examine any potential structural changes, the crystallinity of **5** was examined via PXRD after soaking of the samples for 3 days in the metal nitrate solutions. The crystallinity of **5** is retained in the presence of all metals except Fe^3+^, where the sample becomes completely amorphous (Figure S11). The concentration dependence of Cu^2+^ turn-off sensing was examined, with significant quenching still observed at 0.1 mM concentrations of Cu^2+^ (Figure 7B).

## Conclusions

Through the use of a range of different modulators, a series of six yttrium frameworks with the naphthalene-2,6-dicarboxylate linker have been synthesized, each exhibiting different coordination, solvation, and void space. Unlike transition metal frameworks in which coordination modulation can be used to enhance crystallinity or impart structural properties whilst maintaining topology, the same technique when used with yttrium leads to a range of polymorphs, with the modulator instead acting as a structural director. The resultant frameworks show considerable structural diversity and high thermal stabilities, with framework **5** presenting interesting luminescence properties in the presence of metal nitrate solutions. This technique could unlock a vast myriad of new yttrium frameworks, with this work generating six structures resulting from just one linker, and a seventh recently published using 2,6-difluorobenzoic acid as modulator (Liu et al., 2018). The combination of modulation along with alternative linkers, varying in their functionality, length, and connectivity, could lead to diverse further interesting frameworks.

## Data Availability Statement

The data which underpin this work are available at http://dx.doi.org/10.5525/gla.researchdata.728.

## Author Contributions

RSF and SLG devised the Project. SLG carried out the research, CW carried out crystallographic analysis, and RSF supervised the project. All authors contributed to the writing of the manuscript.

### Conflict of Interest Statement

The authors declare that the research was conducted in the absence of any commercial or financial relationships that could be construed as a potential conflict of interest.
