# Supplementary material for: Uncovering the Structural Diversity of Y(III) Naphthalene-2,6-Dicarboxylate MOFs Through Coordination Modulation
Source: Front Chem. 2019 Jan 31;7:36. doi: 10.3389/fchem.2019.00036 (PMC6365460; doi:10.3389/fchem.2019.00036)
Supplement: Supplementary file 1 [file Table_1.pdf]

frontiers

Supplementary Material

Uncovering the Structural Diversity of Y(III) Naphthalene-2,6-Dicarboxylate MOFs through Coordination Modulation

Sarah L. Griffin, Claire Wilson, and Ross S. Forgan*

*** Correspondence:** Ross S. Forgan: ross.forgan@glasgow.ac.uk

1 General Experimental Remarks

Chemicals and solvents were purchased from Alfa Aesar, Fluorochem, Tokyo Chemical Industry, Sigma-Aldrich, Strem and VWR and used without further purification.

Powder X-Ray Diffraction (PXRD): PXRD measurements were carried out at 298 K using a PANalytical X'Pert PRO diffractometer (λ ($CuK\alpha_1$) = 1.5405 Å) on a mounted zero background sample stage. PXRD patterns were calculated from single crystal structures using Mercury 3.10.3 (Macrae et al., 2008).

Single Crystal X-Ray Diffraction (SCXRD): Data for **1**, **2**, **4** and **6** were collected using a Rigaku 007HF Cu rotating anode generator equipped with Varimax confocal mirrors with an AFC11 goniometer and a HyPix 6000 detector. Data were collected at 100 K through the use of an Oxford Cryosystems cryostream device. Data were collected and processed using CrysAlis PRO 1.171.39.30d (Rigaku OD, 2015).

Data for **3** and **5** were collected using a using a Bruker D8 Venture goniometer with a Bruker PHOTON II detector and dual IμS 3.0 microfocus sources (Cu and MoKα) equipped with an Oxford Cryosystems n-Helix device for collection at 150 K. Data were collected and processed using APEX3 Ver. 2016.9-0 (Bruker, 2016).

Thermogravimetric Analysis (TGA): Measurements were carried out using a TA Instruments Q500 Thermogravimetric Analyser. Measurements were collected from room temperature to 800 °C with a heating rate of 10 °C/min under an air atmosphere.

Nuclear Magnetic Resonance Spectroscopy (NMR): NMR spectra were recorded on either a Bruker AVIII 400 MHz spectrometer or a Bruker AVI 500 MHz spectrometer and referenced to residual solvent peaks

Fluorescence: Fluorescence of **5** was measured on a Shimadzu RF-5301PC spectrofluorimeter with a 150 W xenon lamp, using a medium scan speed and an excitation/emission slit width of 3 nm.

The data which underpin this work are available at http://dx.doi.org/10.5525/gla.researchdata.728.

2 Synthesis

2.1 $[Y_2(NDC)_3(C_3H_7NO)_2]_n$ (1)

Yttrium chloride hexahydrate (0.225 mmol, 0.0439 g) and naphthalene-2,6-dicarboxylic acid (0.225 mmol, 0.0486 g) were added to a 25 mL jar followed by DMF (6 mL). The solution was sonicated until material fully dispersed before placing in the oven at 120 °C for 24 hr. After cooling, the resultant crystals were rinsed several times with fresh DMF and acetone, followed by vacuum drying. Replacing yttrium chloride hexahydrate with yttrium nitrate hexahydrate resulted in the same product, suggesting that counterions do not play a significant structure directing role under these conditions.

The same material could also be synthesised rapidly via microwave assisted heating, using the above quantities and heating for 1 hr at 120 °C in a 35 mL glass vessel. The resultant material underwent the same washing process as for the solvothermal synthesis (Figure S1).

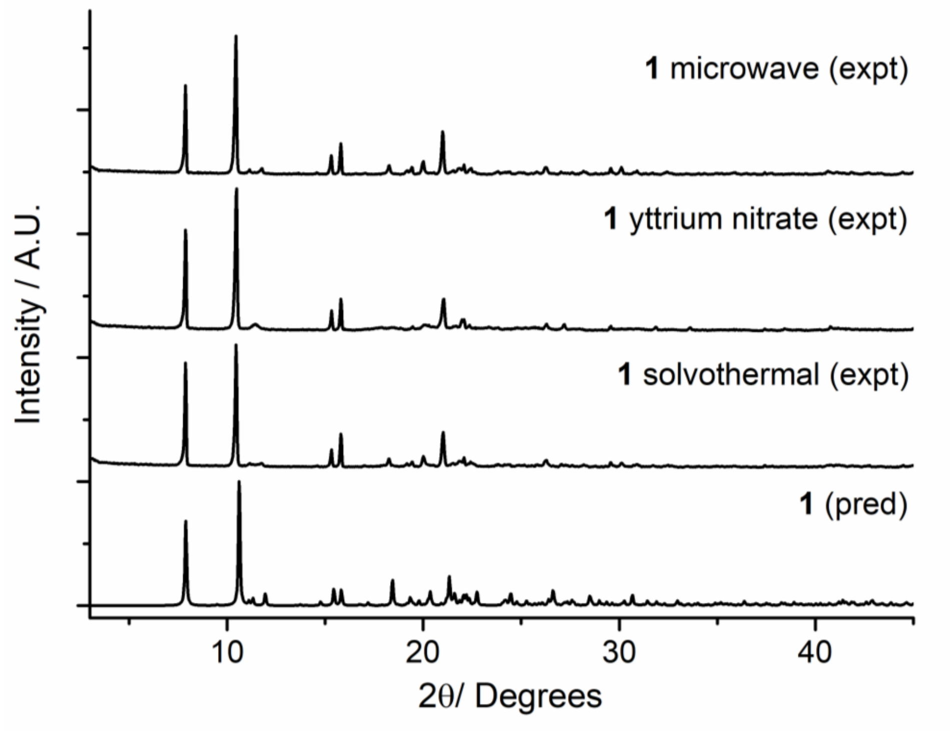

Figure S1. Comparison of the calculated and experimental PXRD patterns of **1**, confirming phase purity via both solvothermal and microwave synthesis, and the ability to produce **1** using yttrium nitrate hexahydrate.

Crystal data for **1**: $C_{42}H_{32}N_2O_{14}Y_2$, M_r = 966.51, crystal dimensions 0.08 x 0.03 x 0.01 mm, Monoclinic, a = 22.8109 (12) Å, b = 8.9656 (4) Å, c = 18.958 (1) Å, α = γ = 90 °, β = 101.180 (5) °, V = 3803.6 (3) Å^3, T = 100 K, space group $P2_1/c$, Z = 4, 13826 measured reflections, 13826 unique (R_{int} = 0.1410) which were used in all calculations. The final R_1 = 0.144 for 9269 observed data $R[F^2 > 2\sigma(F^2)]$ and $wR(F^2)$ = 0.404. Crystal structure data are available from the CCDC, deposition number 1878168.

2.2 [$Y_3(NDC)_4(C_3H_7NO)_4(NO_3)]_n$ (2)

Yttrium nitrate hexahydrate (0.1125 mmol, 0.043 g) and naphthalene-2,6-dicarboxylic acid (0.1125 mmol, 0.0243 g) were added to a 25 mL jar followed by DMF (3 mL) and water (4 µL). The solution was sonicated until material fully dispersed before placing in the oven at 120 °C for 24 hr. After cooling, the resultant selected block crystals were rinsed several times with fresh DMF (Figure S2).

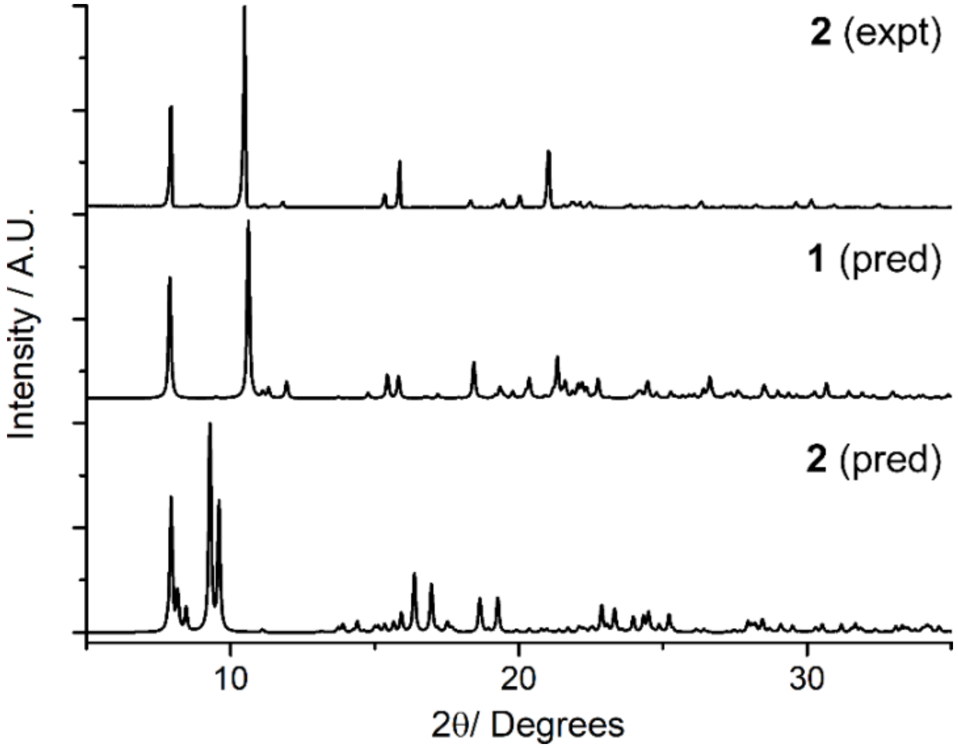

Figure S2. Comparison of the calculated and experimental PXRD patterns of **2**, highlighting the predominant phase of bulk **2** to be **1**.

Crystal data for **2**: $C_{60}H_{52}N_5O_{23}Y_3$, M_r = 1477.79, crystal dimensions 0.1 x 0.003 x 0.01 mm, Triclinic, a = 12.1214 (3) Å, b = 12.6147 (3) Å, c = 12.9711 (3) Å, α = 61.844 (3) °, β = 68.913 (2) °, γ = 80.619 (2) °, V = 1631.53 Å^3, T = 100 K, space group P-1, Z = 1, 29838 measured reflections, 5957 unique (R_{int} = 0.042), which were used in all calculations. The final R_1 = 0.074 for 5653 observed data $R[F^2 > 2\sigma(F^2)]$ and $wR(F^2)$ = 0.227. Crystal structure data are available from the CCDC, deposition number 1878169.

2.3 [$Y_2(NDC)_3(C_3H_7NO)_4]_n$ (3)

Yttrium nitrate hexahydrate (0.225 mmol, 0.086 g) and naphthalene-2,6-dicarboxylic acid (0.225 mmol, 0.0486 g) were added to a 50 mL jar followed by DMF (6 mL), water (15 µL) and nitric acid (150 µL). The solution was sonicated until material fully dispersed before placing in the oven at 120 °C for 24 hr. Upon removal the jar was placed in a cooled room, approximately 16 °C, and left undisturbed for 3 days, resulting in the crystallisation of block crystals which were left in the mother liquor (Figure S3).

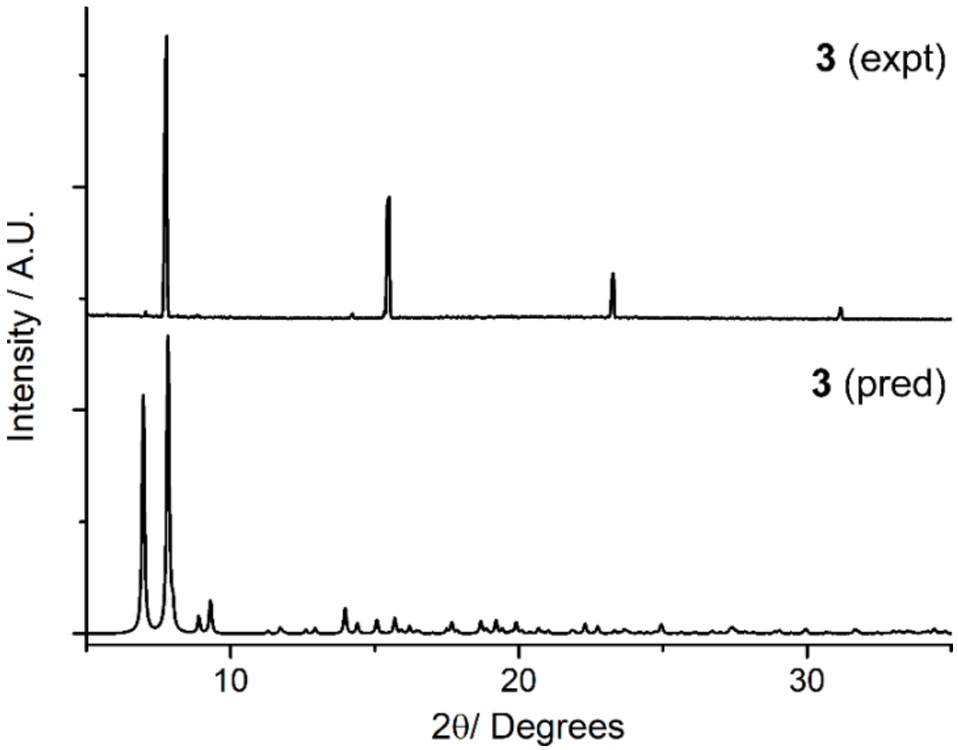

Figure S3. Comparison of the calculated and experimental PXRD patterns of **3**, highlighting the instability of **3** to drying.

Crystal data for **3**: $C_{48}H_{46}N_4O_{16}Y_2$, M_r = 1112.71, crystal dimensions 0.18 x 0.09 x 0.06 mm, Triclinic, a = 11.8479 (7) Å, b = 12.2628 (7) Å, c = 13.3571 (8) Å, α = 102.851 (2) °, β = 98.902 (2) °, γ = 106.554 (2) °, V = 1763.51 (18) $Å^3$, T = 150 K, space group P-1, Z = 1, 17617 measured reflections, 8689 unique (R_{int} = 0.043), which were used in all calculations. The final R_1 = 0.056 for 6358 observed data $R[F^2 > 2\sigma(F^2)]$ and $wR(F^2)$ = 0.153. Approximately 32% of the cell volume is not occupied by the framework and contains diffuse and disordered solvent molecules. This electron density was accounted for using SQUEEZE within PLATON (Spek, 2015) which calculated a solvent accessible volume of 607 $Å^3$ containing 179 electrons (the equivalent of ~3.9 molecules of DMF) per unit cell. Crystal structure data are available from the CCDC, deposition number 1878170.

2.4 [Y(NDC)(CH_3CO_2)(C_3H_7NO)]$_n$ (4)

Yttrium nitrate hexahydrate (0.05625 mmol, 0.0215 g) and naphthalene-2,6-dicarboxylic acid (0.05625 mmol, 0.01215 g) were added to a 25 mL jar followed by DMF (3 mL) and acetic acid (130 μL). The solution was sonicated until material fully dispersed before placing in the oven at 120 °C for 24 hr. After cooling, the resultant plate crystals were rinsed several times with fresh DMF and acetone, followed by vacuum drying.

The material was could also be rapidly synthesised via microwave assisted heating, increasing the quantities by a factor of four, and heating to 120 °C for 2 hr in a 35 mL glass vessel. The resultant material underwent the same washing process as for the solvothermal synthesis. These quantities were also applied for bulk scale solvothermal synthesis (Figure S4).

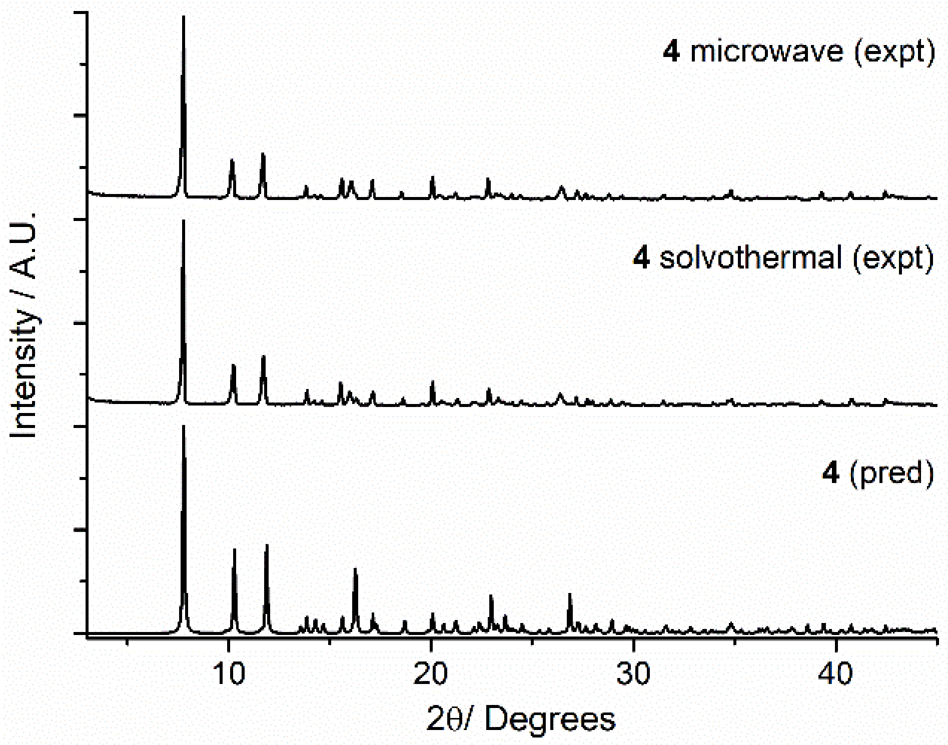

Figure S4. Comparison of the calculated and experimental PXRD patterns of **4**, confirming phase purity via both solvothermal and microwave synthesis.

Crystal data for **4**: $C_{17}H_{16}NO_7Y$, M_r = 435.22, crystal dimensions 0.10 x 0.03 x 0.01 mm, Triclinic, a = 8.7129 (3) Å, b = 9.0506 (2) Å, c = 11.6315 (4) Å, α = 80.722 (2) °, β = 80.807 (3) °, γ = 88.735 (2) °, V = 893.59 (5) Å^3, T = 100 K, space group P-1, Z = 2, 16175 measured reflections, 3231 unique (R_{int} = 0.044), which were used in all calculations. The final R_1 = 0.054 for 3097 observed data $R[F^2 > 2\sigma(F^2)]$ and $wR(F^2)$ = 0.178. Crystal structure data are available from the CCDC, deposition number 1878171.

2.5 $(CH_3)_2NH_2[Y_3(NDC)_3(HCOO)_3(OH)]_n$ (5)

Synthesis conditions were adapted from a previously reported procedure for analogous rare earth MOFs (Xue et al., 2013).

Yttrium nitrate hexahydrate (0.0435 mmol, 0.0167 g), naphthalene-2,6-dicarboxylic acid (0.0435 mmol, 0.0094 g) and 2-fluorobenzoic acid (0.348 mmol, 0.0488 g) were added to a 25 mL jar followed by DMF (2.5 mL), water (0.5 mL) and nitric acid (70 μL). The solution was sonicated until all material fully dispersed before placing in the oven at 120 °C for 24 hr. after cooling, the resultant crystals were washed several times with fresh DMF and acetone, followed by vacuum drying.

The material could also be synthesised rapidly via microwave assisted heating, increasing the quantities by a factor of four and heating to 120 °C for 2 hr in a 35 mL glass vessel. The resultant material underwent the same washing process as for the solvothermal synthesis. These quantities were also applied for bulk scale solvothermal synthesis (Figure S5).

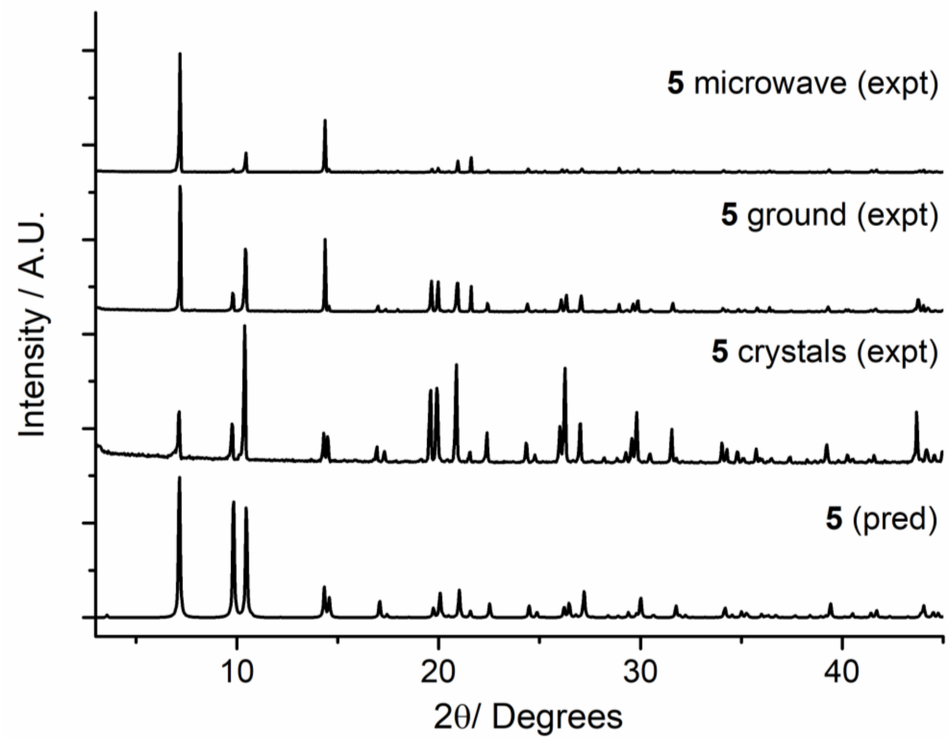

Figure S5. Comparison of calculated and experimental PXRD patterns of **5**, showing preferential orientation of whole crystals compared to ground, and confirming phase purity of material synthesised both solvothermally and via microwave assisted heating.

Crystal data for **5**: $C_{41}H_{30}NO_{19}Y_3$, M_r = 1107.39, crystal dimensions 0.23 x 0.15 x 0.08 mm, Trigonal, a = 10.3765 (17) Å, c = 24.695 (4) Å, $\alpha = \beta = 90$ °, $\gamma = 120$ °, V = 2302.7(8) $Å^3$, T = 150 K, space group P-3, Z = 2, 15960 measures reflections, 3542 unique (R_{int} = 0.064), which were used in all calculations. The final R_1 = 0.064 for 3072 observed data $R[F^2 > 2\sigma(F^2)]$ and $wR(F^2)$ = 0.173. Approximately 20% of the cell volume is not occupied by the framework and contains diffuse and disordered solvent molecules. This electron density was accounted for using SQUEEZE within PLATON (Spek, 2015) which calculated two solvent accessible voids of 188 $Å^3$ and 225 $Å^3$ containing in total 116 electrons (the equivalent of ~1.25 molecules of DMF) per unit cell. Crystal structure data are available from the CCDC, deposition number 1878172.

To prepare the sample for ^{1}H NMR spectroscopy (Figure S6), a small quantity of **5** was added to DMSO-d_6 with five drops of D_2SO_4, followed by heating until all material was solubilised. The integral ratios of characteristic resonances for the aromatic protons of the NDC ligand were compared to those of the in situ generated formate and dimethylammonium species. The integral ratio for NDC:dimethylammonium:formate was found to be 3:1:2.6, which correlates closely to the crystallographically derived ratio of 3:1:3.

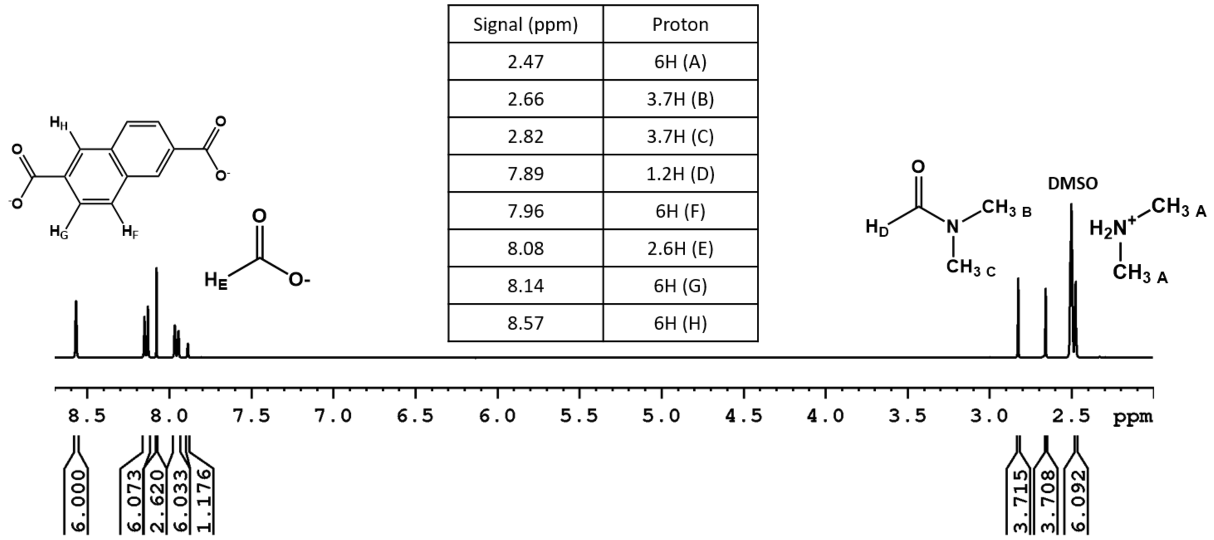

Signal (ppm)	Proton
2.47	6H (A)
2.66	3.7H (B)
2.82	3.7H (C)
7.89	1.2H (D)
7.96	6H (F)
8.08	2.6H (E)
8.14	6H (G)
8.57	6H (H)

Figure S6. ^{1}H NMR spectrum of acid digested (D_2SO_4 / DMSO-d_6) **5** with peak assignments.

2.6 [$Y_2(NDC)_3(C_2H_5O)(H_2O)_3\cdot 3(C_3H_7NO)$] (6)

Yttrium nitrate hexahydrate (0.225 mmol, 0.0862 g), naphthalene-2,6-dicarboxylic acid (0.225 mmol, 0.0486 g) and tartaric acid (0.1125 mmol, 0.0169 g) were added to a 25 mL jar followed by DMF (7.5 mL), water (3.75 mL) and ethanol (3.75 mL). The solution was sonicated before placing in the oven at 80 °C for 24 hr. Single crystals were isolated from the resultant gel. The bulk material was spun down, washing with DMF and acetone, yielding a mixture of phases (Figure S7).

Crystal data for **6**: $C_{47}H_{51}O_{19}NY_2$, M_r = 1139.73, crystal dimensions 0.07 x 0.04 x 0.03 mm, Triclinic, a = 12.4262 (5) Å, b = 12.6835 (5) Å, c = 18.0111 (5) Å, α = 74.078 (3) °, β = 83.157 (3) °, γ = 65.184 (4) °, V = 2477.69 (18) Å^3, T = 100 K, Z = 2, 18517 measured reflections, 18517 unique (R_{int} = 0.0460), which were used in all calculations. The final R_1 = 0.081 for 13777 observed data $R[F^2 > 2\sigma(F^2)]$ and $wR(F^2)$ = 0.270. Three molecules of DMF were modelled within the void space of the framework. Crystal structure data are available from the CCDC, deposition number 1878173.

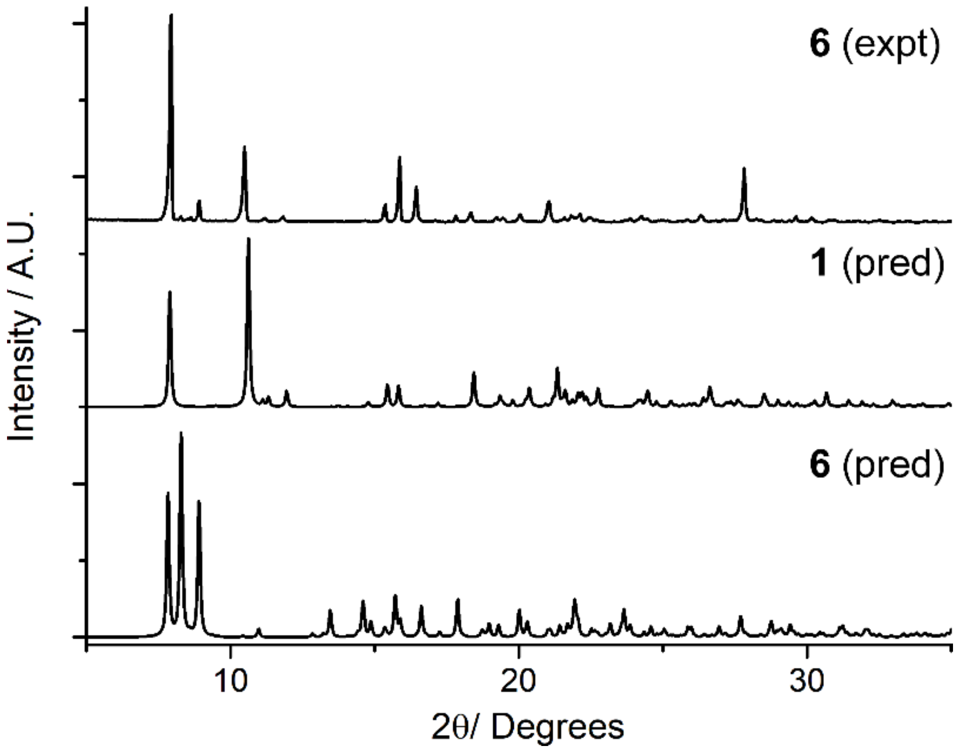

Figure S7. Comparison of the calculated and experimental PXRD patterns of **6**, highlighting the predominant phase of bulk **6** to be **1**.

3 TGA analysis

Thermogravimetric analysis (TGA) was performed on all MOF samples to determine thermal stability. The profiles were collected by heating from room temperature to 800 °C at a rate of 10 °C/min under an air atmosphere (Figures S8 and S9).

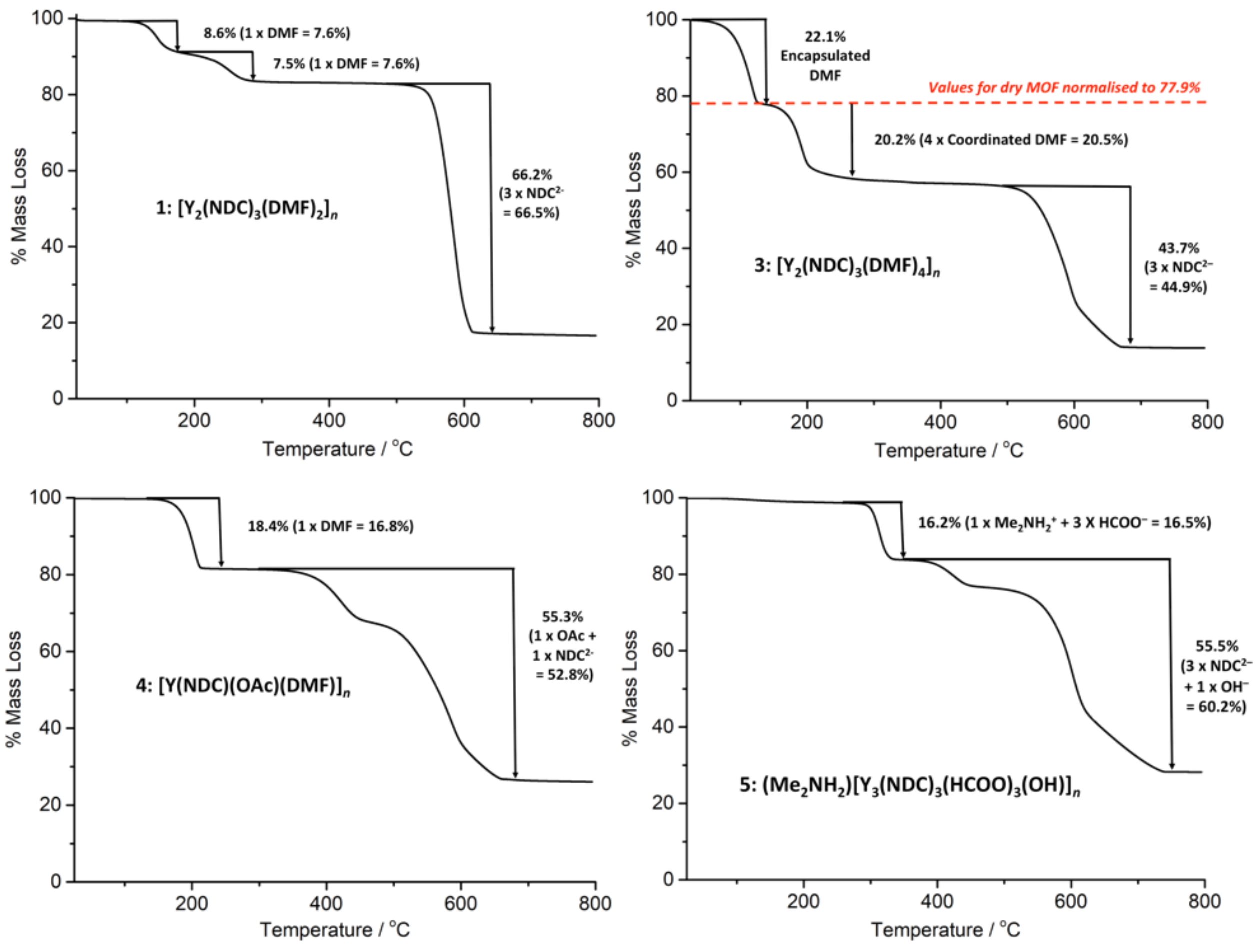

Figure S8. Comparison of TGA profiles of **1**, **3**, **4** and **5**, with assigned weight loss.

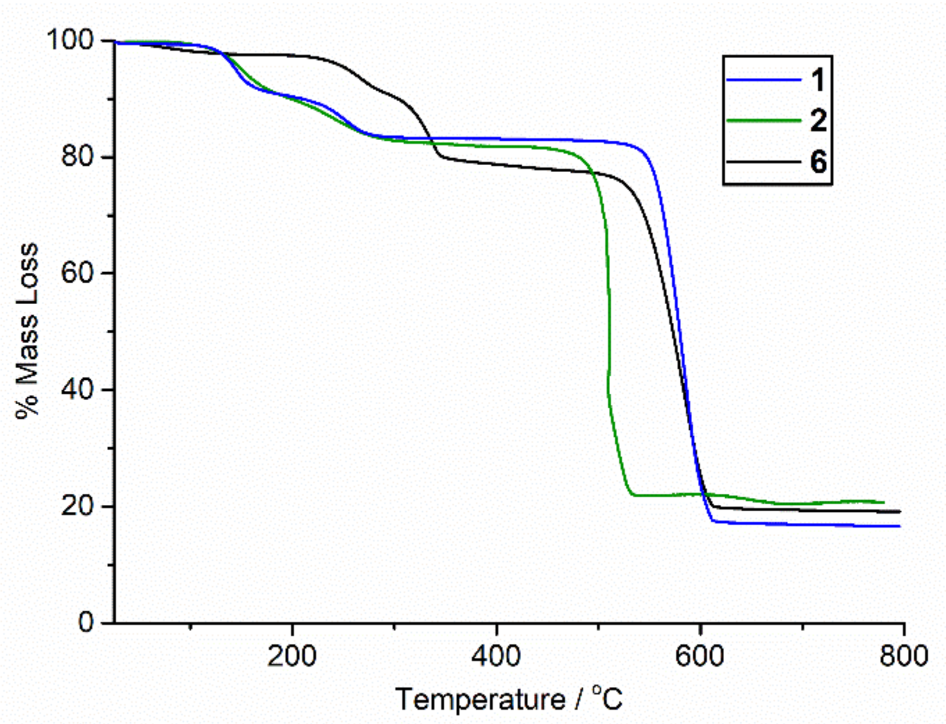

Figure S9. Comparison of the TGA profiles of **2** and **6** to **1**, showing similarities due to the bulk phase being predominantly **1**.

4 Fluorescence Analysis

The MOF sample of **5** used in the fluorescence measurements was prepared via the microwave synthesis method, followed by extensive grinding of the crystalline material to produce a fine powder. The finely ground material (2 mg) was suspended in 5 ml of 5 mM DMF/metal nitrate solutions and sonicated for 30 minutes to ensure suspension before running fluorescence measurements (λ_{ex} = 287 nm, Figure S10).

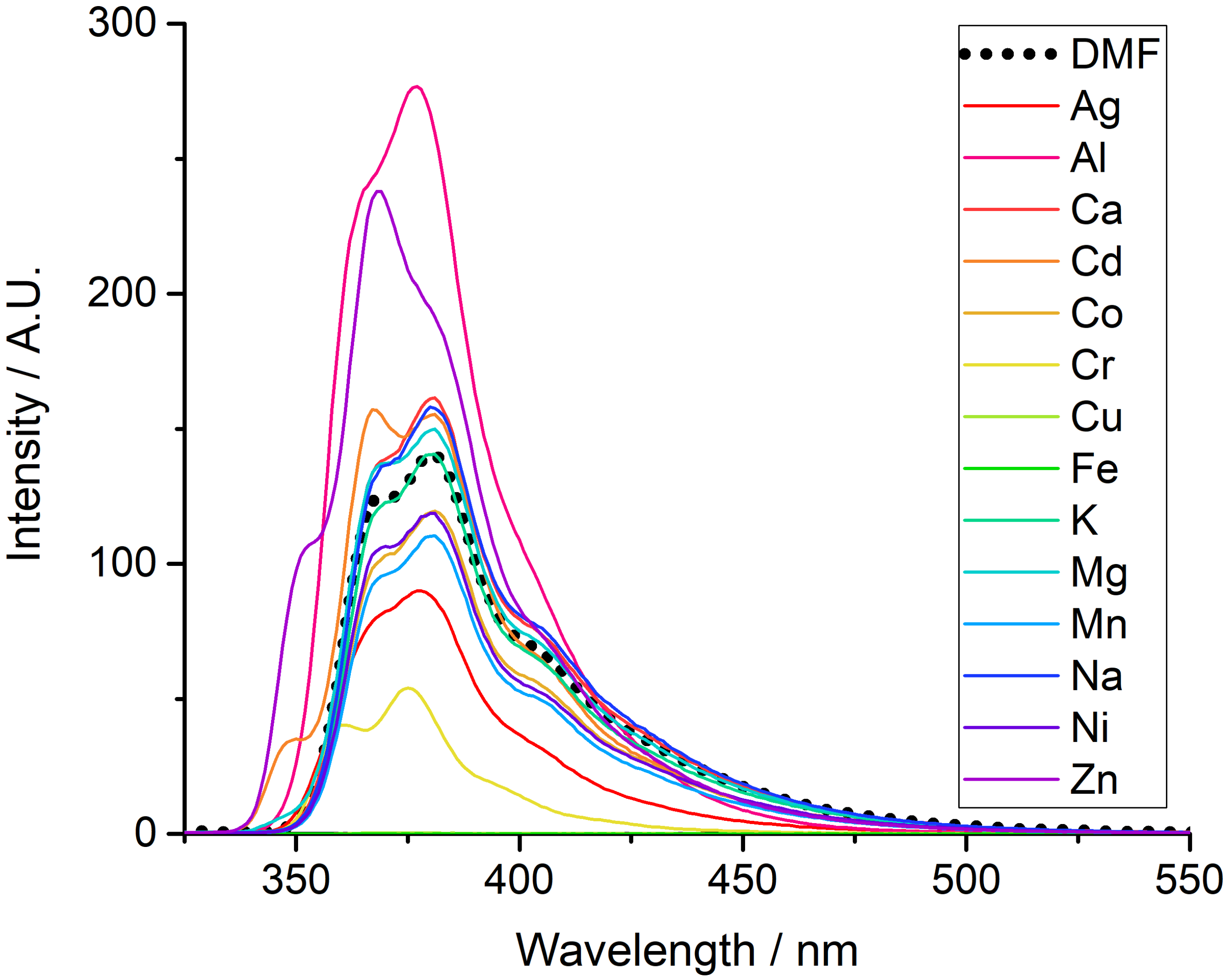

Figure S10. Fluorescence spectra of **5** in the presence of different metal salts (0.005 M, DMF).

The same quantity of **5** was used in the study of Cu^{2+} quenching, suspended in 5 ml solutions of the appropriate concentration. Three days following the measurements, PXRD of the samples was carried out to assess the crystallinity of the material (Figure S11).

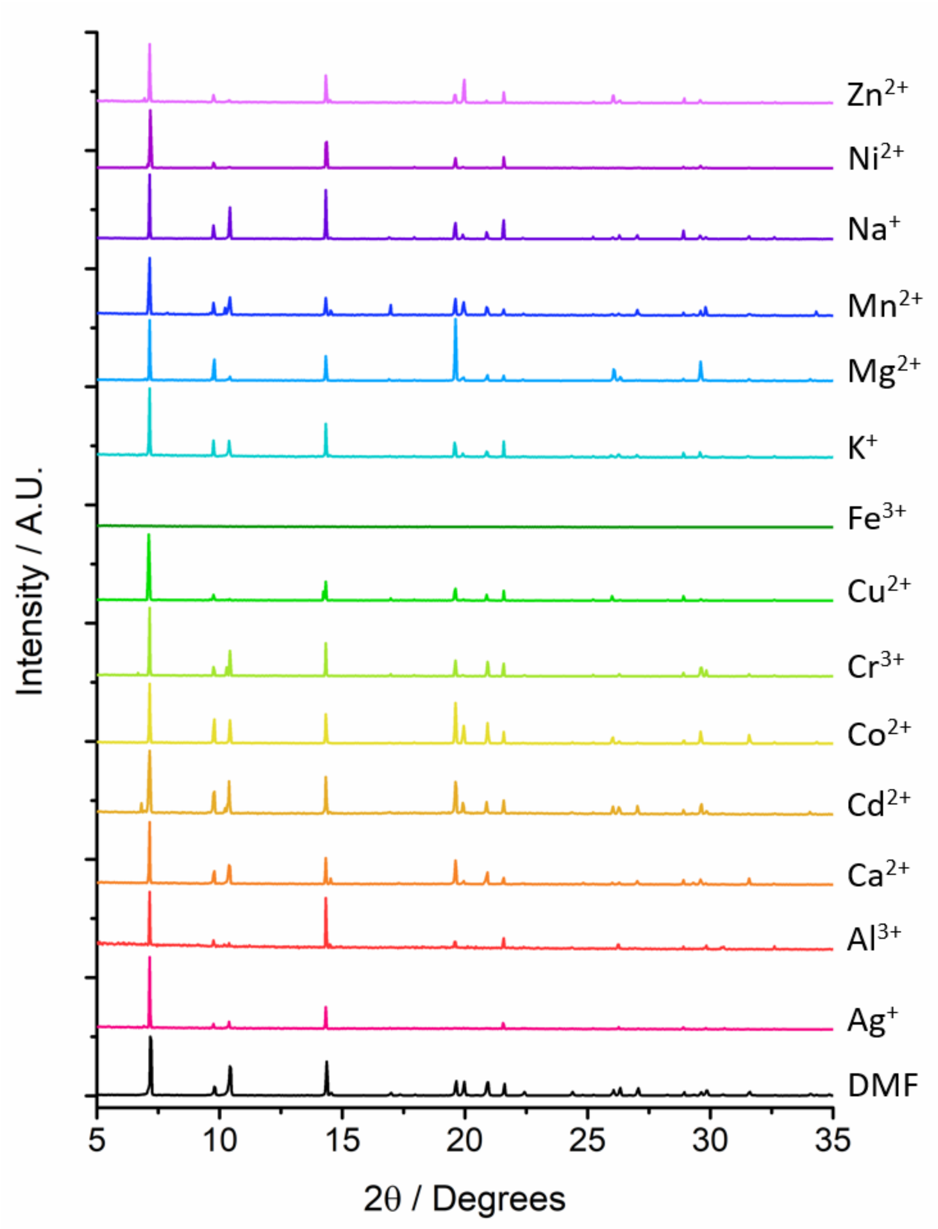

Figure S11. Stacked PXRD patterns of **5** following cation exchange, with all samples showing retention of crystallinity, apart from exposure to Fe^{3+}, which causes the decomposition of **5**.

5 References

Bruker (2016), APEX3, Bruker AXS Inc., Madison, Wisconsin, USA.

Macrae, C. F., Bruno, I. J., Chisholm, J. A., Edgington, P. R., Mccabe, P., Pidcock, E., Rodriguez-Monge, L., Taylor, R., Van De Streek, J., and Wood, P. A. (2008). Mercury CSD 2.0 - new features for the visualization and investigation of crystal structures. *J. Appl. Cryst.* 41, 466-470.

Rigaku OD (2017), CrysAlisPRO, Rigaku Oxford Diffraction, UK.

Spek, A. L. (2015). PLATON SQUEEZE: a tool for the calculation of the disordered solvent contribution to the calculated structure factors. *Acta Cryst. C* 71, 9-18.

Xue, D. -X., Cairns, A. J., Belmabkhout, Y., Wojtas, L., Liu, Y., Alkordi, M. H., and Eddaoudi, M. (2013). Tunable Rare-Earth fcu-MOFs: A Platform for Systematic Enhancement of CO_2 Adsorption Energetics and Uptake. *J Am. Chem. Soc.* 135, 7660-7667.
